# Headache: Treatment update

**DOI:** 10.1016/j.ensci.2022.100420

**Published:** 2022-08-17

**Authors:** Oyindamola I. Ogunlaja, Peter J. Goadsby

**Affiliations:** a NIHR King's Clinical Research Facility and SLaM Biomedical Research Centre, King's College London, UK; b Department of Neurology, University of California, Los Angeles, CA, USA

**Keywords:** Migraine, Treatment, Resource limited settings

## Abstract

Primary headache disorders in particular migraine are one of the most common causes of disability worldwide. Given the high burden of migraine in terms of disability, there has been an effort to develop migraine specific therapies that has led to the availability of new drugs including 5HT_1F_ receptor agonists-ditans (lasmiditan), small molecule calcitonin gene-related peptide (CGRP) receptor antagonists-gepants: (ubrogepant, rimegepant, atogepant) and anti-CGRP monoclonal antibodies (erenumab, fremanezumab, galcanezumab and eptinezumab).

However, some of these treatments incur a high cost and may not be a feasible option for most patients in resource limited settings. Lasmiditan and the gepants are a good option for patients with moderate-severe migraine attacks who cannot use triptans due variously to poor tolerability, or cardio- or cerebrovascular disease. For practical purposes, the new anti-CGRP monoclonal antibodies are best reserved for patients who have failed to have efficacy or had intolerable side effects from multiple traditional oral preventives.

## Highlights

• Newly available treatments for migraine include the ditans (lasmiditan), gepants and anti-CGRP monoclonal antibodies.
• Lasmiditan is a good option for acute treatment in patients who have contraindications to triptans.
• CGRP monoclonal antibodies are good preventive treatment options for patients who have failed standard oral preventives.

## 1 Introduction

Primary headache disorders in particular migraine are one of the most common causes of disability worldwide. In 2016 [1] almost 3 billion people worldwide were estimated to have migraine or tension-type headache −1.89 billion with tension-type headache and 1.04 billion with migraine, however migraine caused 45.1 million years of life lived with disability (YLDs) in comparison to tension-type headache with 7.2 million YLDs globally in 2016 [1]. Similarly, a high burden of migraine was found in Africa [1].

Given the high burden of migraine in terms of disability, there has been an effort to develop migraine specific therapies that has led to the availability of new drugs including 5HT_1F_ receptor agonists-ditans (lasmiditan), small molecule calcitonin gene-related peptide (CGRP) receptor antagonists- gepants: (ubrogepant, rimegepant, atogepant) and anti-CGRP monoclonal antibodies (erenumab, fremanezumab, galcanezumab and eptinezumab). However, some of these treatments incur a high cost in the range of thousands of US dollars and may not be a feasible option for most patients in resource limited settings. The developments have offered insights for the future, such as vasoconstriction is not required as a mechanism for an acute migraine therapy, and ultimately the promise of small molecule generics.

Broadly, the treatment strategy for migraine is divided into acute and preventive therapies. Acute therapies refer to medication typically used acutely to ameliorate symptoms, while preventive therapies are designed to be taken to reduce the frequency or severity of migraine, or both.

## 2 Acute migraine treatment

Acute therapies range from simple analgesics such as non-steroidal anti-inflammatory drugs (NSAIDs), acetaminophen (paracetamol) to the migraine specific triptans: serotonin 5-HT_1B/1D_ receptor agonists and ergot derivatives [2]. Migraine is often accompanied by nausea and vomiting that can be as bothersome and disabling as the pain, hence antiemetics, such as metoclopramide, domperidone, ondansetron and prochlorperazine are often essential components of abortive regimens. While various NSAIDs have shown efficacy in randomized placebo-controlled trials of migraine therapy [3] their use is limited by potential gastrointestinal and renal side effects. They are also not effective in all patients with migraine.

### 2.1 Triptans

Triptans were developed in the 1980s [4]. They act on components the pathophysiological mechanism of migraine by inhibiting the release of vasoactive peptides, promoting vasoconstriction, and inhibiting pain pathways in the brain stem [2].

Oral triptans have comparable efficacy to NSAIDs, acetaminophen, and aspirin and are likely superior to ergots: necessarily this is a population-studied assertion. Specifically, with standard dose triptans, 18–50% of patients had freedom from pain at 2 h compared with placebo (11%) whereas NSAIDs (22%), acetaminophen (22%), and ergots (16%) were associated with less favorable outcomes [5]. In particular, sumatriptan subcutaneous injection (37%), eletriptan tablets (39%), rizatriptan orally disintegrating tablet (ODT) (50%), and zolmitriptan ODT (37%) were associated with the most favorable outcomes using placebo-correction as the metric [5]. All traditional oral triptans are now available generically and hence should be generally affordable.

Given the vasoconstrictive properties of triptans, they are contraindicated in individuals with a history of ischemic heart disease, stroke, uncontrolled hypertension, hemiplegic migraine, and peripheral vascular disease due to their theoretical risk of worsening these conditions.

### 2.2 Ditans

Lasmiditan is a selective 5HT_1F_ receptor agonist that acts on the trigeminal system without causing vasoconstriction due to its low affinity for 5HT_1B_ receptors [6]. This makes it a suitable alternative for patients with relative contraindications to triptans due to cardiovascular risk factors. In a randomized trial [7] of 2231 patients, significantly more patients given lasmiditan 200 mg (32.2%, *P* < 0.001) and 100 mg (28.2%, *P* < 0.001) were headache pain free at 2 h after the first dose compared with patients who received placebo (15.3%). In a second trial in patients with migraine with and without aura, the percentage of patients who were headache pain free at 2 h was statistically significant compared to placebo across all dosing groups: for 50 mg (28.6%, *P* = 0.003); for 100 mg (31.4%, *P* < 0.001); for 200 mg (38.8%, *P* < 0.001) and 21.3% for placebo [8].

It was approved by the US Food and Drug Administration (FDA) in 2019 based on the results of these two trials. It is available in 50 mg and 100 mg oral tablets. The initial dose is 50 or 100 mg and there is no benefit to taking a second dose for the same migraine attack so it should be taken only once in 24 h [9]. The most commonly reported side effects in trials were dizziness, paresthesia and somnolence. It also causes impairment in driving performance with a driving simulator study showing impairment at 1.5 h post dose but not at 8 h [10]. Therefore, patients who take it are advised not to drive or perform potentially hazardous activities for at least 8 h post dose. These side effects limit its use in clinical practice for patients who drive or for some occupations. It is best considered for patients who have contraindications or inadequate response to triptan use and who's migraine onset occurs in the evenings.

### 2.3 Gepants

Extensive research supporting the role of calcitonin gene-related peptide (CGRP) in the pathophysiology of migraine led to the development of several drugs targeting the CGRP peptide and receptor [11]. To date, there have been two oral small molecule CGRP receptor antagonists (gepants) approved by the FDA for the acute treatment of migraine – ubrogepant and rimegepant.

Ubrogepant) was approved by the FDA in December 2019 with two randomized controlled clinical trials showing efficacy [12,13]. Adults with migraine, with or without aura were assigned in a 1:1:1 ratio to receive an initial dose of placebo, ubrogepant 50 mg or ubrogepant 100 mg for the treatment of a single migraine attack. The primary endpoints were pain freedom at 2 h and absence of most bothersome migraine associated symptom at 2 h. The percentage of patients who were pain free at 2 h was 11.8% in the placebo group, 19.2% in patients who received ubrogepant 50 mg, and 21.2% in the 100 mg ubrogepant group (*P* < 0.001). In terms of freedom from the most bothersome symptom at 2 h, the percentages were 27.8% in the placebo group, 38.6% in the 50 mg ubrogepant group, and 37.3% in the 100 mg ubrogepant group. The most common side effects were nausea, somnolence, and dry mouth [12]. Long-term intermittent use of ubrogepant 50 and 100 mg for the acute treatment of migraine was safe and well tolerated as determined by an open label 52-week extension trial [14].

Rimegepant similarly received FDA approval for the acute treatment of migraine in February 2020. It is available in a 75 mg orodispersable tablet with three randomized, phase 3, double blind placebo-controlled trials showing efficacy and safety of the drug. At 2 h post dose rimegepant was superior to placebo for freedom from pain (21% vs 11%, *P* < 0.0001) [15].In the another phase 3 trial with an oral formulation, the percentage of patients who were pain free at 2 h after receiving rimegepant was 19.6% in comparison to 12% in the placebo group (*P* < 0.001) [16].In a third, Rimegepant was again superior to placebo for freedom from pain at 2 h post dose (19.2% vs 14.2%, *P* < 0.03) [17]. The most common adverse events were nausea and urinary tract infection. CGRP receptor antagonism does not cause vasoconstriction making gepants a reasonable alternative for patients who cannot take triptans due to cardiovascular disease [18].

In 2015, the American headache society (AHS) published guidelines for the acute treatment of migraine [19]. A updated consensus statement was also recently published to guide the use of the new acute and preventive treatments available for migraine [20]. It would be reasonable to begin with the simple over the counter nonspecific medications for patients with mild to moderate attacks and reserve triptans, ditans and gepants for moderate-severe attacks or mild to moderate attacks that do not respond to non-specific therapy.

## 3 Preventive treatment of migraine

Drugs used for the preventive treatment of migraine come from many classes. Prior to the development of the medications targeting the CGRP ligand/receptor there was only one relatively specific migraine preventive medication – methysergide, which is no longer available anywhere in the world due to serious adverse events: retroperitoneal fibrosis [21].

In 2012, a guideline [22] was developed by the American Headache Society (AHS) and the American Academy of Neurology (AAN) that assessed the level of efficacy of available preventives. The following have Level A evidence (i.e., established efficacy with two high quality studies supporting efficacy)– topiramate, metoprolol, propranolol, divalproex sodium, botulinum toxin A. Amitriptyline received a level B rating (i.e., probably effective) due to insufficient completion rates in the included trials. All these oral medications are readily available and relatively inexpensive and should therefore be the first option for patients in low resource settings. The choice of which to start should be individualized to each patient based on their comorbid medical conditions, side effect profile, the patient's preferences, and individual characteristics (e.g., in females of reproductive age, divalproex sodium should be avoided as it is highly teratogenic).

On average, approximately 50–60% of patients given any of these medications will have a 50% reduction in headache frequency, and the doses required for this effect often lead to intolerable side effects [23,24].

### 3.1 CGRP pathway monoclonal antibodies

There are currently four CGRP monoclonal antibodies available for the preventive treatment of migraine. Erenumab, eptinezumab, fremanezumab and galcanezumab approved in various parts of the world including by the US FDA and the European Medicines Agency. Erenumab specifically targets the canonical CGRP receptor while the other three are antibodies against the CGRP ligand itself. They all have superior efficacy to placebo [[25], [26], [27], [28], [29], [30], [31], [32], [33], [34]] with similar population efficacy rates to traditional oral preventives for migraine [35]. When compared head-to-head with topiramate, erenumab was superior in tolerability and efficacy rate [36]. No head-to-head comparisons have been done between the different antibodies, but they appear to have similar efficacy to each other with a therapeutic gain range of 22–23.7% across all the antibodies [37].

The CGRP monoclonal antibodies are generally well tolerated, which contrasts sharply with traditional oral preventives, with the most common adverse events being injection site and hypersensitivity reactions. Constipation and hypertension have also been reported with erenumab. Long term safety data is available for erenumab in the form of information from the open label treatment phase of a randomized control trial showing no increase in the incidence of adverse events, serious adverse events or adverse events leading to the discontinuation of treatment over 5 years of exposure to the drug [38]. CGRP is a potent vasodilator therefore, there are theoretical concerns that CGRP antagonism could precipitate or worsen ischemic events in patients with vascular disease by inhibiting compensatory vasodilation. However, a pooled analysis of migraine prevention studies found no increased incidence of vascular adverse events in patients taking erenumab [39]. One caveat being that patients who had major vascular events in the 12 months prior were excluded from the clinical trials. Thus, the safety of erenumab in high-risk groups remains unknown. The European Headache Federation has published guidelines [40] on the use of CGRP monoclonal antibodies and suggests avoiding their use in patients with cardio- and cerebrovascular disease. It should also not be used in the treatment of women who are pregnant or breastfeeding as there is no data to guide it's use in this population.

These antibodies have demonstrated efficacy in patients who have failed multiple traditional oral preventives in the past [[41], [42], [43]]. Therefore, given their high cost– they are best reserved for these patients.

### 3.2 Gepants

Rimegepant, which was previously mentioned, has also been approved for the preventive treatment of migraine. In a clinical trial with 1591 participants [44], patients with migraine were randomized to receive either rimegepant 75 mg every other day or placebo for 12 weeks. Rimegepant was superior to placebo in terms of the primary endpoint of change in the mean number of migraine days per month during weeks 9–12 of the study. There was a change of −4.3 days with rimegepant and − 3.5 days with placebo (least squares mean difference − 0·8 days, 95% CI -1·46 to −0·20; *P* = 0·0099). Tolerability of the drug was similar to placebo (36% of patient who received rimegepant reported an adverse event, compared with 36% who received placebo). No unexpected or serious adverse events were noted.

Atogepant was approved by the FDA for prevention of migraine in September 2021 based on the results of 2 clinical trials showing effectiveness of the medication. In a double-blind phase 2b/3 trial [45], adults with 4–14 migraine days a month were randomized to receive placebo or atogepant 10 mg once daily, 30 mg once daily, 60 mg once daily, 30 mg twice daily or 60 mg twice daily. The primary outcome was change from baseline in monthly migraine days across the 12-week treatment period. All five atogepant groups showed significant reduction from baseline in mean monthly migraine days vs placebo: atogepant 10 mg once daily −4·0 (0·3; *P* = 0·024), 30 mg once daily −3·8 (0·2; *P* = 0·039), 60 mg once daily −3·6 (0·2; *P* = 0·039), 30 mg twice daily −4·2 (0·4; *P* = 0·0034), and 60 mg twice daily −4·1 (0·3; *P* = 0·0031); placebo −2·9 (0·2). The drug was generally well tolerated with the most common side effect being nausea (range of 5% of patients on the lowest dose of 10 mg once daily to 12% of patients on 60 mg once daily vs 5% for placebo).

In the phase 3 double blind ADVANCE trial [46], adults with 4–14 migraine days per month were randomly assigned in a 1:1:1:1 ratio to receive a once daily dose of 10 mg, 30 mg or 60 mg of oral atogepant or placebo. The primary outcome was change in mean monthly migraine days from baseline across the 12 weeks. The changes from baseline were − 3.7 days with 10 mg of atogepant, −3.9 with 30 mg atogepant, −4.2 with 60 mg atogepant and − 2.5 with placebo. The mean differences from placebo in the change from baseline were − 1.2 days with 10 mg atogepant (95% confidence interval [CI], −1.8 to −0.6), −1.4 days with 30 mg atogepant (95% CI, −1.9 to −0.8), and − 1.7 days with 60 mg atogepant (95% CI, −2.3 to −1.2) (*P* < 0.001 for all comparisons with placebo). A key secondary endpoint measured the proportion of patients that achieved a ≥ 50% reduction in monthly migraine days - 55.6%/58.7%/60.8% of patients in the 10 mg/30 mg/60 mg atogepant groups, respectively achieved at least a 50% reduction, in comparison to only 29% of patients taking placebo (all atogepant groups vs. placebo, *P* ≤ 0.0001). The most common adverse events were constipation and nausea.

## 4 Conclusion

In low resource settings, greater emphasis on lifestyle modification including the benefits of exercise, adequate hydration and nutrition and sleep hygiene may be an inexpensive, albeit less evidence-based, method of reducing the burden of primary headache disorders such as migraine [47]. There are published guidelines/consensus documents [19,20,22,40] available to aid clinicians in the choice of available acute and preventive treatment options for migraine. The new CGRP pathway blockers are perhaps best reserved for patients who have failed to have efficacy or had intolerable side effects from multiple traditional oral preventives, while in terms of acute treatment - lasmiditan and the gepants are a good option for patients with moderate-severe migraine attacks who cannot use triptans due to poor tolerability, or cardio- or cerebrovascular disease.

## Declaration of Competing Interest

OIO has nothing to report.

PJG reports, over the last 36 months, grants and personal fees from Eli-Lilly and Company, grant from Celgene, and personal fees from Aeon Biopharma, Allergan/Abbvie, Amgen, Biodelivery Sciences International, Biohaven Pharmaceuticals Inc., CoolTech LLC, Dr. Reddys, Epalex, Impel Neuropharma, Lundbeck, Novartis, Praxis, Sanofi, Satsuma and Teva Pharmaceuticals, and personal fees for advice through Gerson Lehrman Group, Guidepoint, SAI Med Partners, Vector Metric, and fees for educational materials from CME Outfitters, Omnia Education, WebMD, and publishing royalties or fees from Massachusetts Medical Society, Oxford University Press, UptoDate and Wolters Kluwer, and for medicolegal advice in headache, and a patent magnetic stimulation for headache (No. WO2016090333 A1) assigned to eNeura without fee.
